# A detailed overview of quercetin: implications for cell death and liver fibrosis mechanisms

**DOI:** 10.3389/fphar.2024.1389179

**Published:** 2024-05-23

**Authors:** Fei Xiong, Yichen Zhang, Ting Li, Yiping Tang, Si-Yuan Song, Qiao Zhou, Yi Wang

**Affiliations:** ^1^ Department of Gastroenterology, Sichuan Academy of Medical Science and Sichuan Provincial People’s Hospital, Chengdu, China; ^2^ Department of Rheumatology and Immunology, Sichuan Provincial People’s Hospital, University of Electronic Science and Technology of China, Chengdu, China; ^3^ Department of Rheumatology, Wenjiang District People’s Hospital, Chengdu, China; ^4^ Baylor College of Medicine, Houston, TX, United States; ^5^ Clinical Immunology Translational Medicine Key Laboratory of Sichuan Province, Sichuan Provincial People’s Hospital, University of Electronic Science and Technology of China, Chengdu, China

**Keywords:** quercetin, cuproptosis, liver fibrosis, apoptosis, necroptosis, autophagy, pyroptosis, ferroptosis

## Abstract

**Background:**

Quercetin, a widespread polyphenolic flavonoid, is known for its extensive health benefits and is commonly found in the plant kingdom. The natural occurrence and extraction methods of quercetin are crucial due to its bioactive potential.

**Purpose:**

This review aims to comprehensively cover the natural sources of quercetin, its extraction methods, bioavailability, pharmacokinetics, and its role in various cell death pathways and liver fibrosis.

**Methods:**

A comprehensive literature search was performed across several electronic databases, including PubMed, Embase, CNKI, Wanfang database, and ClinicalTrials.gov, up to 10 February 2024. The search terms employed were “quercetin”, “natural sources of quercetin”, “quercetin extraction methods”, “bioavailability of quercetin”, “pharmacokinetics of quercetin”, “cell death pathways”, “apoptosis”, “autophagy”, “pyroptosis”, “necroptosis”, “ferroptosis”, “cuproptosis”, “liver fibrosis”, and “hepatic stellate cells”. These keywords were interconnected using AND/OR as necessary. The search focused on studies that detailed the bioavailability and pharmacokinetics of quercetin, its role in different cell death pathways, and its effects on liver fibrosis.

**Results:**

This review details quercetin’s involvement in various cell death pathways, including apoptosis, autophagy, pyroptosis, necroptosis, ferroptosis, and cuproptosis, with particular attention to its regulatory influence on apoptosis and autophagy. It dissects the mechanisms through which quercetin affects these pathways across different cell types and dosages. Moreover, the paper delves into quercetin’s effects on liver fibrosis, its interactions with hepatic stellate cells, and its modulation of pertinent signaling cascades. Additionally, it articulates from a physical organic chemistry standpoint the uniqueness of quercetin’s structure and its potential for specific actions in the liver.

**Conclusion:**

The paper provides a detailed analysis of quercetin, suggesting its significant role in modulating cell death mechanisms and mitigating liver fibrosis, underscoring its therapeutic potential.

## 1 Introduction

**FIGURE 1:**
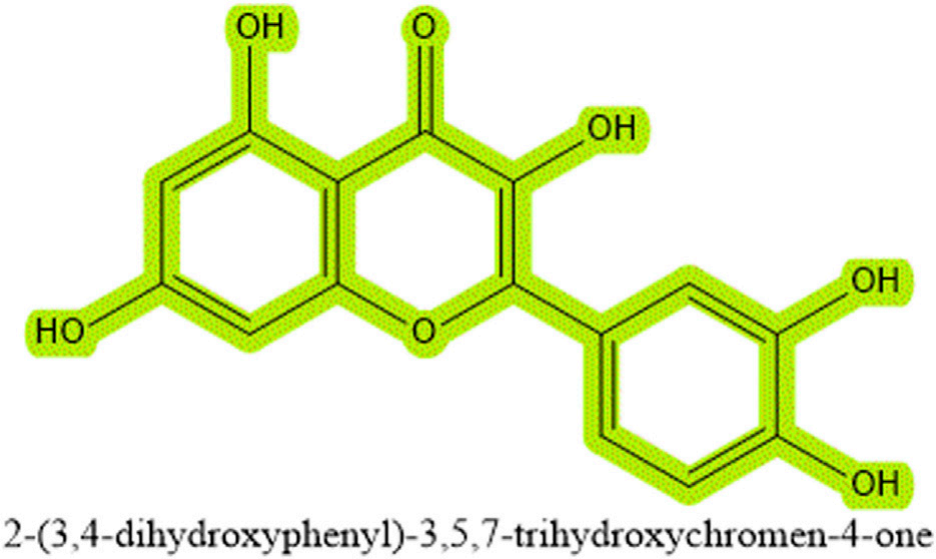
The chemical structure of quercetin (C_15_H_10_O_7_).

Quercetin is a plant-derived flavonol, belonging to the broad category of polyphenolic substances and is ubiquitous in nature (Balestrini et al., 2021). Its etymology traces back to 1857, derived from the Latin “Quercus,” meaning “oak forest” or an area abundant with oaks (Batiha et al., 2020). These metabolites, characterized by a basic C6-C3-C6 diphenylpropane structure, are prevalent in the plant kingdom. Among them, quercetin stands out due to its widespread presence across various plant species. It often forms glycosides when bonded with sugar groups, which endows it with water solubility for easier absorption while maintaining the hydrophobic properties of phenolic substances, although it also exists in its aglycone form, such as in quercetin and kaempferol (García Díaz et al., 2022). As a fundamental flavonoid compound, quercetin forms the structural basis of several other flavonoid compounds like rutin, hesperidin, and naringenin (Rahvar et al., 2018), all sharing a core chemical framework with only minor variations in substituents. From a nomenclature perspective, quercetin is named 2-(3,4-dihydroxyphenyl)-3,5,7-trihydroxy-4H-chromen-4-one, with the chemical formula C15H10O7, accurately depicting its molecular structure (Li et al., 2016) (see Figure 1). Due to the unique chemical characteristics of the benzene ring and the balance between hydrophobicity and hydrophilicity brought by hydroxyl groups and sugar bindings, it plays a significant role in regulating cell death pathways and improving liver fibrosis. Quercetin has been found to participate in various cell death pathways such as apoptosis, autophagy, necrosis, and more, particularly playing a critical role in regulating apoptosis and autophagy processes. Additionally, quercetin has shown significant efficacy in treating liver fibrosis, especially impacting hepatic stellate cells. Moreover, the bioavailability and pharmacokinetics of quercetin are discussed in detail, including its sources and extraction methods. In summary, this article elaborates on the origins, extraction methods, and bioavailability of quercetin, emphasizing its specific mechanisms in intervening various cell death modalities (apoptosis, autophagy, necrosis, pyroptosis, ferroptosis, cuproptosis) and explores its specificity in action mechanisms from a physical organic chemistry perspective, especially concerning the liver. Further discussions include the impact of quercetin on liver fibrosis.

## 2 Methods of data acquisition

To ensure a comprehensive and systematic review of the existing literature on quercetin, a meticulous search strategy was implemented. Several electronic databases were queried for relevant publications, including PubMed, Embase, CNKI, Wanfang database, and ClinicalTrials.gov. The time frame for the literature search was up to and including 10 February 2024, to capture the most recent studies available.

The search terms were carefully selected to encompass the broad spectrum of research areas relevant to quercetin. Keywords included “quercetin”, “natural sources of quercetin”, “quercetin extraction methods”, “bioavailability of quercetin”, “pharmacokinetics of quercetin”, “cell death pathways”, “apoptosis”, “autophagy”, “pyroptosis”, “necroptosis”, “ferroptosis”, “cuproptosis”, “liver fibrosis”, and “hepatic stellate cells”. Boolean operators (AND/OR) were employed to combine these terms effectively and to ensure the search was both comprehensive and precise.

The inclusion criteria for the studies were as follows: 1) studies that report on the bioavailability and pharmacokinetics of quercetin, 2) research investigating the role of quercetin in various cell death pathways, and 3) studies focusing on the effects of quercetin on liver fibrosis. Studies were excluded if they did not directly pertain to these areas or if they were not in the English language. The search was not restricted by study design to capture a wide range of methodologies, from *in vitro* experiments to clinical trials. Upon retrieval, records were screened based on titles and abstracts to identify those that met the inclusion criteria. Full-text articles were then obtained and further assessed for relevance. Data extraction focused on the methods of quercetin extraction, its pharmacokinetic profile, and detailed accounts of its role in cellular pathways leading to cell death, with a special focus on liver fibrosis. The extracted data was then synthesized and prepared for comprehensive analysis within the review.

## 3 Results

### 3.1 Origin and therapeutic potential of quercetin

#### 3.1.1 Quercetin sources

Quercetin is a polyphenolic flavonoid produced by plants through the phenylpropanoid pathway, a complex process initiated from cinnamic acid that encompasses eight distinct steps (Nabavi et al., 2020). This metabolites, serving as a secondary metabolite, enjoys a broad distribution across the plant world, predominantly in the form of glycosides, which are quercetin molecules bound to sugar molecules (Morimoto et al., 2023). A wide array of foodstuffs features quercetin, making it easily obtainable from dietary sources. Notable examples include onions, capers, various berries, apples, grapes, vegetables of the Brassica genus, tea, shallots, tomatoes, and an assortment of seeds, nuts, barks, flowers, and leaves. Medicinal botanical drugs such as *Hypericum perforatum* (St. John’s Wort), Ginkgo biloba, and Sambucus canadensis (Elderberry) also contain quercetin (Wiczkowski et al., 2008; Li et al., 2016).

The quercetin content significantly differs among foods, with raw capers being particularly rich in quercetin, boasting 234 mg per 100 g of the edible part. Conversely, teas like black or green tea from Camellia sinensis are on the lower end, with a mere 2 mg per 100 g (Bhagwat et al., 2011). The extraction of quercetin from plant sources has evolved, moving from traditional methods such as Soxhlet extraction, which is known for its solvent-heavy, time-consuming, and less efficient yield process, to more modern techniques (M et al., 2023; Gligor et al., 2023). Techniques such as microwave-assisted extraction (MAE) and supercritical fluid extraction (SFE) are now preferred for their efficiency and rapid processing times (Belwal et al., 2018).

As a metabolites with a vast pharmacological profile, quercetin is often incorporated into dietary supplements, available in powder and capsule forms. Consumption levels of quercetin vary globally, influenced by the intake of fruits, vegetables, and tea, with daily intakes estimated between 50 and 800 mg. For example, average quercetin consumption has been reported at 18 mg in China, 16.2 mg in Japan, 18.48 mg in Spain, and 9.75 mg in the United States (Li et al., 2016). In summary, quercetin’s widespread presence in various plant-based foods, coupled with advancements in extraction methodologies, has enhanced its accessibility and utility. Its varied dietary sources and established health benefits position quercetin as a valuable nutrient within the realms of nutrition and healthcare.

#### 3.1.2 Bioavailability and pharmacokinetics of quercetin

The burgeoning interest in quercetin’s health benefits has catalyzed the production of nutraceuticals containing high doses of this metabolites (Naghibi et al., 2013). Nonetheless, the challenge of quercetin’s low oral bioavailability, particularly after a single dose due to macronutrient absorption barriers, remains significant (Rich et al., 2017). Human pharmacokinetic studies have pinpointed the oral bioavailability of quercetin at a mere 2% following a single dose (Li et al., 2016), a limitation often ascribed to its slow absorption, quick metabolism, and swift bodily elimination. The bioavailability variance of quercetin metabolites is notably influenced by their sugar moiety (Graefe et al., 2001), with quercetin glycosides exhibiting more efficient human absorption than the aglycone form (Wagner et al., 2006). The absorption efficiency also varies with the type of sugar attached to the quercetin molecule (Scholz and Williamson, 2007), further moderated by the dietary matrix and presence of other dietary factors like fiber and fat (Guo et al., 2013).

In plant sources, quercetin primarily exists as hydrophilic glycosides, which can be converted into the aglycone form by β-glucosidases in the small intestine, facilitating passive diffusion through the intestinal epithelium (Ader et al., 2000). This process is possibly aided by the intestinal sodium/glucose cotransporter-1, allowing direct bloodstream entry of quercetin glycosides (Murota and Terao, 2003). Inside the body, quercetin may be oxidized, forming derivatives such as quercetin-quinone and quercetin-quinone methides (Rietjens et al., 2005; van der Woude et al., 2005). Unabsorbed quercetin undergoes colonic microbiota degradation, producing phenolic acids that are absorbed and transported to the liver for further conjugation (Manach et al., 2005; Thilakarathna and Rupasinghe, 2013).

Post-absorption, quercetin is metabolized in enterocytes and the liver through glucuronidation, sulfation, and methylation, generating a spectrum of metabolites (van der Woude et al., 2004; Harwood et al., 2007). The primary metabolites include isorhamnetin and glucoside acid-sulfated derivatives, constituting 91.5% of all quercetin metabolites, alongside glucuronoside and methylated variants (Morand et al., 1998). The primary metabolites include isorhamnetin and glucoside acid-sulfated derivatives, constituting 91.5% of all quercetin metabolites, alongside glucuronoside and methylated variants (de Boer et al., 2005), with notable deconjugated quercetin presence in certain tissues, albeit with variations in free quercetin proportions across different organs (Bieger et al., 2008). The distribution of quercetin aglycone in tissues, however, remains uncertain due to potential postmortem deconjugation variances (de Boer et al., 2005). Some studies propose mitochondrial quercetin accumulation, though evidence is still emerging (Moon et al., 2008; Konrad and Nieman, 2015).

Quercetin’s urinary concentration escalates with intake dosage and time post-consumption, highlighting the kidneys’ role in its excretion (Young et al., 1999; Mullen et al., 2006). Metabolic by-products include 3-hydroxyphenylacetic acid, hippuric acid, and benzoic acid (Guo and Bruno, 2015), with liver-produced metabolites potentially being expelled via the biliary tract through MRP2 (Arts et al., 2004; Guo and Bruno, 2015) and from the lungs in high quantities (Li et al., 2016). Quercetin elimination is protracted, with half-lives ranging between 11 and 28 h, and an average terminal half-life of approximately 3.5 h (Konrad and Nieman, 2015). Research by Ferry et al. on intravenous quercetin in cancer patients identified a safe dosage at 945 mg/m^2, contrasting with higher doses that induced adverse effects such as emesis and nephrotoxicity (Hollman and Katan, 1998). Graefe et al.'s study maintained a dosage up to 200 mg, observing a Cmax of 2.3 ± 1.5 μg/mL and a Tmax of 0.7 ± 0.3 h [39], underscoring the metabolite pharmacokinetic complexity.

### 3.2 Quercetin's influence on cellular death mechanisms

Cell death occurs through various pathways, including apoptosis, autophagy, necroptosis, pyroptosis, ferroptosis, and cuproptosis, each with distinct triggers and processes (refer to Table 1). Quercetin, a versatile flavonoid, has shown efficacy in influencing these diverse cell death pathways. (Table 2 provides detailed listings of the dosages, models, and treatment durations related to quercetin mentioned in the cited literature).

**TABLE 1:** Overview of cell death modes, inducing factors, and Quercetin’s targets.

Modes of cell death	Classification	Inducing factor	Target for quercetin
Apoptosis	- Intrinsic pathway: BAX and BAK regulate mitochondrial dysfunction; Release of mitochondrial cytochrome c and changes in mitochondrial membrane potential; Regulation of E2F1 and p53 proteins in the cell nucleus	Tumor necrosis factor (TNF), DNA damage, oxidative stress, and mitochondrial dysfunction	ROS, PI-3-kinase, MCL, Bcl-2, BCL-XL, ER stress, BAD, BAX, cyclooxygenase
	- Extrinsic pathway: Overexpression of TRAMP, TRAIL receptor-mediated signaling pathway		
Autophagy	- Macroautophagy: mTOR pathway, ULK1ATG13FIP200 complex, ULK1-AMPK pathway, formation of the autophagosome membrane by phosphorylating and activating Beclin-1, VPS34, and autophagy-related genes (ATG) 14, ATG12-ATG5-ATG16L1, and LC3-PE conjugation systems	Nutrient deprivation or energy stress	mTOR, LC3-PE, AMPK, TFEB
	- Microautophagy: Hsc70, KFERQ, LAMP-2A		
	- Chaperone-mediated autophagy: The endosomal sorting complex required for transport (ESCRT)		
Necroptosis	none	Physical trauma, infection, or exposure to toxins	RIPK1, IKKα and Iκbα phosphorylation, NO
Pyroptosis	- Canonical pathway: activation of caspase-1	Infection or endogenous challenge	NLRP3 inflammasome, LPS/ATP
	- Noncanonical pathway: caspase-4/5 and caspase-11		
Ferroptosis	none	Metabolic dysfunction of intracellular lipid oxidation, iron	Iron overload, FPN, the uptake of O2, soybean lipoxygenase-I-dependent linoleic acid peroxidation, ATF3
Cuproptosis	none	Copper toxicity	Cells heavily dependent on mitochondrial metabolism, copper, chelate copper ions, intestinal copper metabolism

**TABLE 2:** Overview of cell death modes, inducing factors, and Quercetin’s targets.

Document title	Dosage range of quercetin	Model used
Quercetin induces tumor-selective apoptosis	10–40 µM	Human leukemia U937 cells
Quercetin stimulates mitochondrial apoptosis	0.5–120 μM	Primary rat hepatic stellate cells (HSCs)
The role of activated MEK-ERK pathway in quercetin	14.5–58.0 μM	Human A549 lung carcinoma cells
Quercetin induces cytochrome-c release	10–150 μM	Cervical carcinoma HeLa cells
Quercetin induces apoptosis in hepatoma cell line (HepG2)	10–100 mmol/L	Human hepatoma HepG2 cells
Quercetin protects retina from oxidative stress injury	5–80 µM	Human retinal pigment epithelial cells
Enhancing TFEB-Mediated Cellular Degradation by Quercetin	0.5–20 μM	Retinal pigment epithelial (RPE) cells
Quercetin induces autophagy-associated death in HL-60	25, 50 μM	Acute myeloid leukemia (AML) cell lines
Quercetin against Type 2 Diabetes in muscle cells	10 and 100 μM	L6 rat skeletal muscle cells
Quercetin attenuates NF-kappaB in rat hepatocytes	5–100 μmol/L	Rat hepatocytes
Quercetin alleviates intestinal injury in weaned pigs	10 μmol/L	Weaned pigs and porcine epithelial IPEC-1
Quercetin on macrophage pyroptosis	0–100 μM	Human monocytic THP-1 cells
Quercetin Inhibits Inflammasome Activation	Cell culture and mouse doses	Mouse vasculitis model and BMDM cells
Quercetin inhibits IL-18 secretion in keratinocytes	5 and 10 mM	Human primary and HaCaT keratinocytes
Quercetin–iron chelates and glucose transporters	0.1μM–10 μM	MDCK canine kidney epithelial cells
Quercetin Alleviates Ferroptosis in Type 2 Diabetes	1.5 g/kg in mice	High-fat diet-induced type 2 diabetic mice
Iron-overload induces oxidative DNA damage	6.25–100 μM	Human colon carcinoma HT29 clone 19A
Quercetin inhibits intestinal iron absorption	50 mg/kg in rats; 0.1–10 mmol/L	Caco-2 cells and rat models
Quercetin alleviates acute kidney injury	25 mg/kg in mice; 10 μM	Mice and NRK-52E, HK-2 cells
Quercetin enhances copper induction of metallothionein	3–10µM–100 µM	Human intestinal Caco-2 cells
Inhibitory effects of quercetin on liver fibrosis	5 and 15 mg/kg	Rat liver fibrosis induced by CCl4
Quercetin prevents hepatic fibrosis	100 mg/kg and 200 mg/kg	BDL and CCl4 models in mice
Effect of Daily Ingestion of Quercetin-Rich Onion Powder	9 g of onion powder	Healthy Japanese subjects, BMI 23–30
Phase I Dose Escalation Study of Quercetin	250 mg–5000 mg	Patients with chronic hepatitis C

#### 3.2.1 Quercetin and apoptosis

Apoptosis is a form of programmed cell death characterized by specific morphological and biochemical changes such as cell shrinkage, membrane blebbing, chromatin condensation, DNA fragmentation, and caspase activation (Shi, 2004). It is driven by two main pathways: the intrinsic (mitochondrial) and the extrinsic (death receptor) pathways (Harada, 2003). The intrinsic pathway is activated by internal stimuli including DNA damage, oxidative stress, and mitochondrial dysfunction, leading to the release of cytochrome c from mitochondria. This release triggers a cascade that activates caspase-9, followed by caspase-3, culminating in cell death (Wei et al., 2001; Meyer et al., 2005; Dewson and Kluck, 2009; Gupta et al., 2009; Parrish et al., 2013). The extrinsic pathway, on the other hand, begins when external ligands bind to death receptors on the cell surface, activating caspase-8, which then activates caspase-3, leading to apoptosis (Bodmer et al., 1997; Stennicke HR et al., 1998; Guicciardi and Gores, 2009).

**FIGURE 2:**
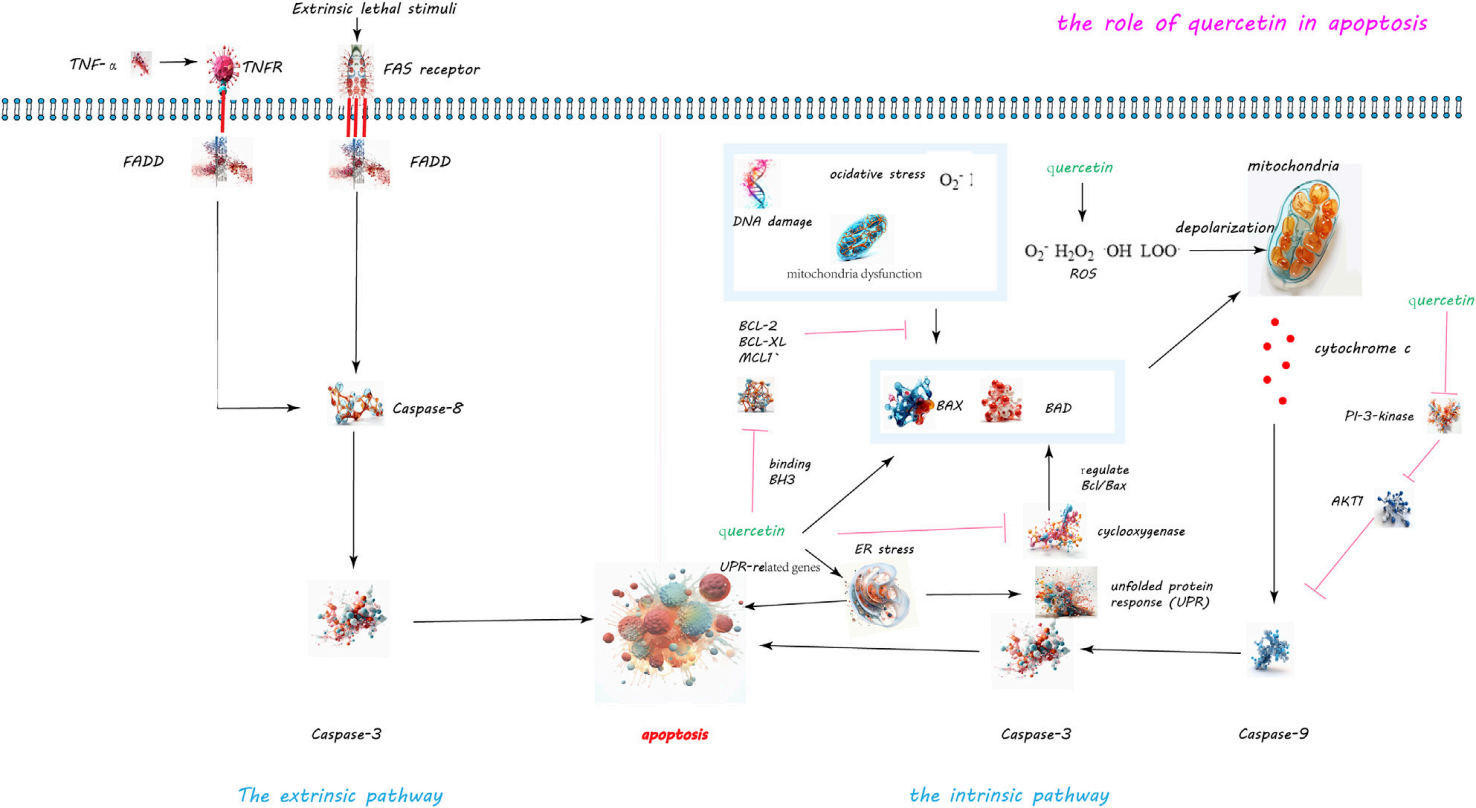
Quercetin-Induced Apoptotic Pathways in Cellular Regulation. The dual apoptotic pathways are within cells: the intrinsic and extrinsic pathways. The intrinsic pathway, activated by internal signals such as DNA damage or mitochondrial disruptions, leads to the activation of caspase-9 and caspase-3. Conversely, the extrinsic pathway is initiated by the external binding of death ligands to cellular death receptors, catalyzing the activation of caspase-8. Quercetin’s role in inducing apoptosis is highlighted through its capacity to disrupt the balance between anti-apoptotic and pro-apoptotic proteins. Specifically, quercetin downregulates the anti-apoptotic protein Mcl-1 and activates the pro-apoptotic protein Bax, while also inhibiting the activity of survival proteins Bcl-2 and Bcl-xL. Moreover, quercetin’s induction of reactive oxygen species (ROS) precipitates the release of cytochrome c and diminishes mitochondrial membrane potential, paving the way for apoptosis. Additionally, quercetin triggers endoplasmic reticulum stress and obstructs the PI3K/Akt signaling pathway, further augmenting apoptotic processes.

Quercetin has been identified as a potent inducer of apoptosis in various cancer cell types including breast, prostate, lung, colon, and leukemia cells, as evidenced by its ability to disrupt the balance between pro-apoptotic and anti-apoptotic proteins (see Figure 2). In an experiment conducted by Cheng et al., mice were administered quercetin intraperitoneally at doses of 0, 20, and 40 mg/kg body weight daily for 15 consecutive days. Researchers observed that in leukemia cells treated with quercetin, the expression of the anti-apoptotic protein Mcl-1 was downregulated, while the pro-apoptotic protein Bax was activated and translocated to the mitochondrial membrane. Furthermore, the extent of these effects was found to increase with the dosage of quercetin, indicating a dose-dependent relationship (Cheng et al., 2010). Studies have observed that quercetin concentrations above 20 μM significantly inhibit the proliferation of hepatic stellate cells (HSCs) with an IC50 of 27.2 μM, but it only affects liver cell growth at concentrations up to 80 μM (IC50 of 68.5 μM). Quercetin stimulates apoptosis in HSCs, with an apoptosis rate reaching 40% at a concentration of 40 μM (EC50 of 51.6 μM). Quercetin reduces the expression of Bcl-2, increases the expression of Bax, and leads to the release of cytochrome C within cells. Additionally, quercetin also enhances the mRNA and protein expression of calnexin and CHOP in HSCs, but does not affect liver cells. Moreover, quercetin increases the phosphorylation of PERK and IRE1 and the cleavage of ATF6. However, the ER stress inhibitor alubrinal significantly reduces the apoptotic effect of quercetin induced in HSCs (He et al., 2016).

Quercetin’s apoptotic effect in A549 lung cancer cells includes increasing the levels of Bax and Bad while decreasing Bcl-2, with the rate of apoptosis rising in correlation with the concentration of quercetin. The study employed four different concentrations of quercetin: 14.5, 29.0, 43.5, and 58.0 μM, with treatment durations of 24 and 48 h. The results indicate that the apoptosis rate increases as the concentration of quercetin increases (Nguyen et al., 2004). Quercetin triggers the production of reactive oxygen species (ROS), mitochondrial membrane depolarization, and the release of cytochrome c, demonstrating its capability to initiate apoptosis through mitochondrial pathways. The concentrations of quercetin used in this study ranged from 30 to 90 μM (Bishayee et al., 2013). Quercetin induces apoptosis in fetal rat hepatocytes by enhancing ROS production, leading to the loss of mitochondrial transmembrane potential, cytochrome c release, and caspase-3 activation. The study found that an 18-h treatment with quercetin can induce apoptosis in HepG2 cells, while a shorter treatment duration (4 h) has no effect on cell viability. Moreover, the inducing effect of quercetin is dose-dependent. Therefore, the specific dosage of quercetin needs to be determined based on the circumstances (Granado-Serrano AB et al., 2006).

Furthermore, quercetin induces endoplasmic reticulum (ER) stress by increasing UPR-related gene expression and promoting PERK and IRE1 phosphorylation, thereby activating apoptosis in hepatic stellate cells (He et al., 2016). It also inhibits the PI3K/Akt signaling pathway, reducing Akt phosphorylation and consequently inactivating procaspase-9, which plays a critical role in apoptosis regulation (Gamet-Payrastre et al., 1999; Granado-Serrano AB et al., 2006). Additionally, quercetin activates the ERK pathway in a dose- and time-dependent manner, influencing apoptosis by potentially promoting caspase-3 activation (Nguyen et al., 2004). Recent studies have shown that quercetin at concentrations of 10, 20, or 30 µM can significantly reduce the viability of HepG2 cells, especially with the treatment of 30 µM quercetin. This treatment also significantly lowers the mitochondrial membrane potential, demonstrating quercetin’s ability to induce apoptosis in HepG2 cells. Flow cytometry experiments have proven that quercetin induces apoptosis in a dose-dependent manner. This effect occurs through the direct interaction of quercetin with YY1, disrupting the YY1-p53 interaction, promoting p53 activation, and leading to an increased Bax/Bcl-2 ratio (Guan et al., 2023). Quercetin also has great potential in the prevention and treatment of liver cancer. By regulating multiple signaling pathways, including upregulating Bax, caspase-3, and p21, and downregulating Akt, PLK-1, cyclin-B1, cyclin-A, CDC-2, CDK-2, and Bcl-2, quercetin has been shown to induce apoptotic cell death in liver cancer cells (Sethi et al., 2023). In summary, quercetin modulates cell death by altering the equilibrium between pro- and anti-apoptotic proteins, generating oxidative stress, disrupting mitochondrial function, and inducing ER stress. It also affects key signaling pathways like PI3K/Akt and MAPK/ERK, contributing to its apoptotic effects. This evidence underscores quercetin’s potential as an anti-cancer agent, with future research needed to further elucidate its mechanisms and optimize its therapeutic applications.

#### 3.2.2 Quercetin’s engagement with autophagy pathways

**FIGURE 3:**
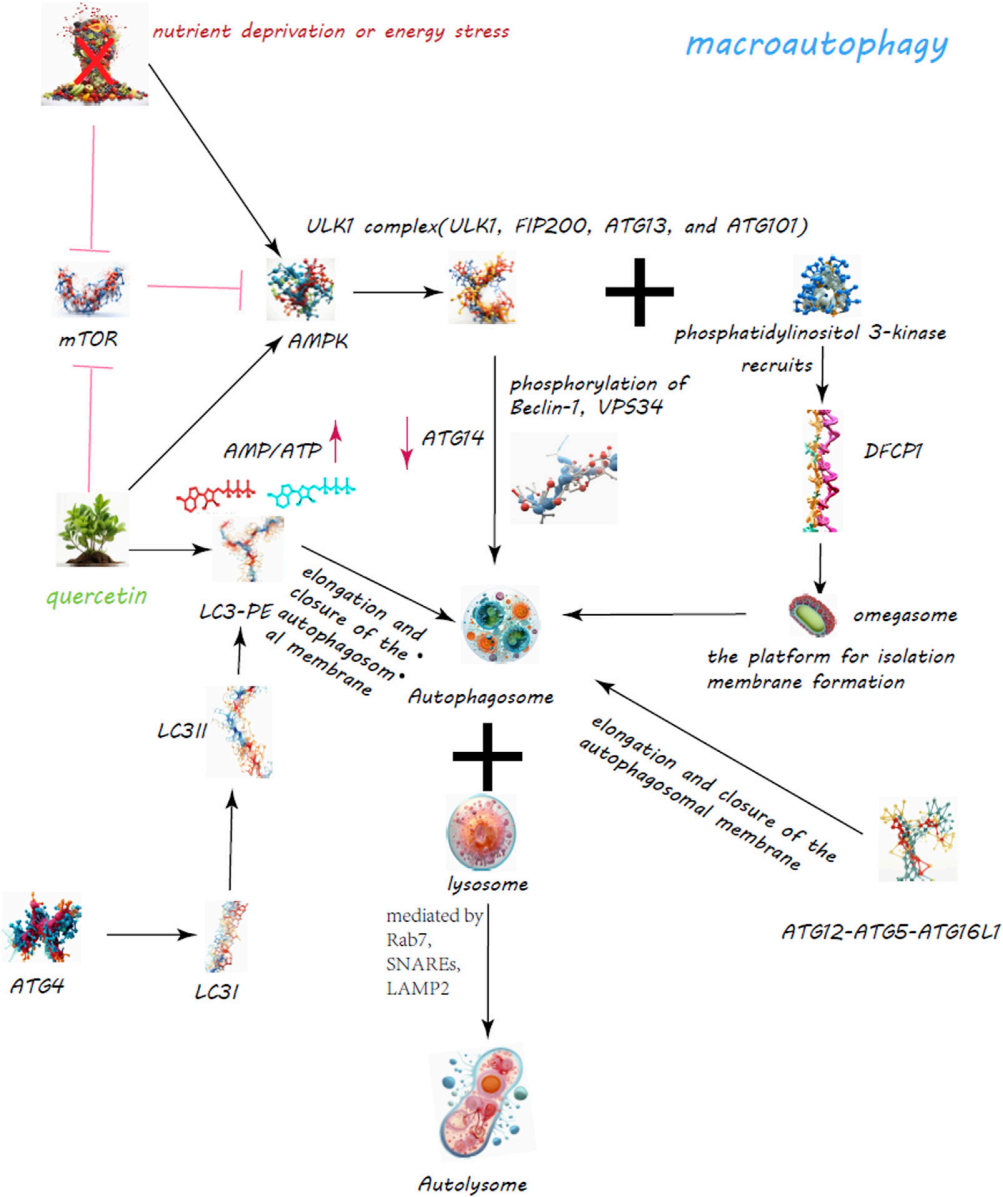
Quercetin’s Enhancement of Macroautophagy for Cellular Regulation. The quercetin’s dynamic role in bolstering macroautophagy is a critical cellular process for maintaining homeostasis through the degradation and recycling of proteins and organelles. Quercetin modulates the mechanistic target of rapamycin (mTOR) pathway and activates AMP-activated protein kinase (AMPK), leading to decreased mTOR activity and the initiation of autophagy.

**FIGURE 4:**
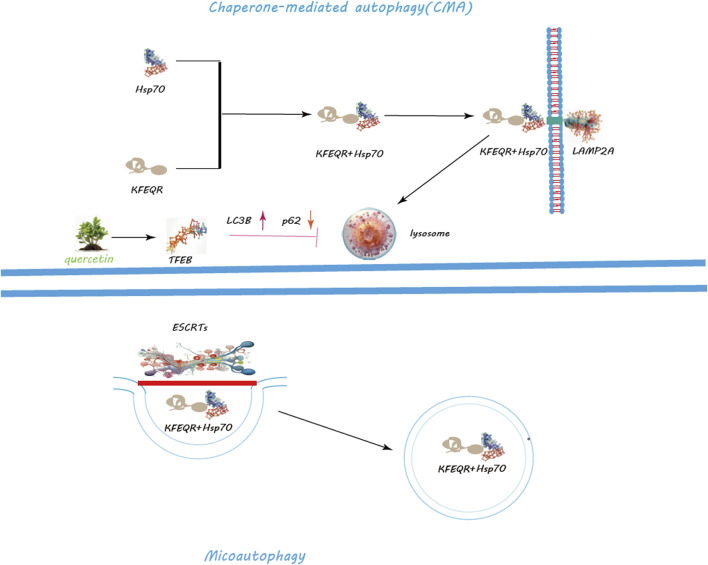
Regulation of CMA and Macroautophagy by Quercetin. Quercetin notably promotes lysosomal biogenesis, crucial for producing lysosomes that contain the enzymes needed for breaking down cellular metabolite s. Quercetin triggers the nuclear translocation of Transcription Factor EB (TFEB), a key regulator of lysosomal biogenesis and autophagy genes, thus amplifying autophagic activity for improved cellular clearance. Additionally, quercetin modulates the mechanistic target of rapamycin (mTOR) pathway and activates AMP-activated protein kinase (AMPK), leading to decreased mTOR activity and the initiation of autophagy. Through these actions, quercetin serves as a potent facilitator of cellular maintenance and health, highlighting its significant impact on enhancing autophagy for cellular equilibrium and stress management.

Autophagy, a critical cellular mechanism for the degradation and recycling of cytoplasmic components, plays a pivotal role in maintaining cellular equilibrium, adapting to stress, and ensuring cellular vitality (Takeshige K et al., 1992). This process manifests in three primary forms: macroautophagy, microautophagy, and chaperone-mediated autophagy (CMA), each tailored to specific cellular needs and degradation targets (Abdrakhmanov A and Zhivotovsky, 2020) (Figures 3, 4).

Macroautophagy, the most studied form, involves the creation of autophagosomes, a process orchestrated by the ULK1 complex, which comprises ULK1, FIP200, ATG13, and ATG101 (Ganley et al., 2009) (Figure 3). Activation of this complex is modulated by AMPK in response to nutrient scarcity or energy stress, and by mTORC1, a nutrient-sensitive regulator (Egan et al., 2011; Egan et al., 2011). Following activation, the ULK1 complex initiates autophagosome formation by phosphorylating key proteins like Beclin-1, VPS34, and ATG14 (Nazarko and Zhong, 2013; Russell et al., 2013; Wold et al., 2016). PI3K activity at the ER generates PI3P, facilitating the recruitment of DFCP1 to form the omegasome, which aids in membrane formation (Dooley et al., 2014; Nascimbeni et al., 2017; Birgisdottir Å et al., 2019). WIPI and ATG2A/B play roles in sourcing membranes via ATG9A for the burgeoning autophagosome (Lystad et al., 2019). Two ubiquitin-like systems, the ATG12-ATG5-ATG16L1 complex and LC3-PE conjugation, govern membrane expansion and closure (Tanida et al., 2004; Bento et al., 2016). LC3 processing involves ATG4, converting LC3-I to its membrane-bound form, LC3-II (Maruyama and Noda, 2017). Fusion of autophagosomes with lysosomes, a step facilitated by Rab7, SNAREs, and LAMP2, completes the degradation process (Bucci et al., 2000; Liu et al., 2020; Tian et al., 2021). CMA targets specific proteins for lysosomal degradation, identified by a KFERQ motif and mediated by Hsc70, leading to their translocation across the LAMP-2A receptor (Majeski and Dice, 2004) (Figure 4). Microautophagy involves direct engulfment of cargo by lysosomes, with the ESCRT machinery playing a role in cargo selection and membrane invagination (Frankel et al., 2017; Avalos-Padilla et al., 2021) (Figure 4).

Quercetin has been observed to stimulate autophagy in various models and conditions, including diabetic nephropathy (Lu et al., 2015), intervertebral disc degeneration (Wang et al., 2020), and retinal pigment epithelial cells under oxidative stress (Li et al., 2021). It exerts effects through modulation of the mTORC1 pathway, promoting autophagy as a protective mechanism against cellular stress and damage.

In studies, quercetin administration showed dose-dependent effects on autophagy induction. For diabetic nephropathy, high doses (90 mg/kg/d) significantly reduced renal fibrosis, mediated through the mTORC1/p70S6K pathway affecting epithelial-mesenchymal transition in tubular epithelial cells (Lu et al., 2015). In retinal cells, quercetin alleviated oxidative stress, promoting the accumulation of the tight junction protein ZO-1 and upregulating autophagy markers such as Beclin-1 and LC3-II. Experiments showed that in ARPE-19 cells, quercetin concentrations of 5 μM and 80 μM were not non-toxic, whereas concentrations of 10 μM, 20 μM, 40 μM, and 60 μM were found to enhance cell viability. Although the clinical application of quercetin is still limited by its low water solubility, poor bioavailability, and extensive first-pass metabolism, new formulations with clinical potential have been proposed. Further research is needed to explore the autophagy-inducing process of quercetin and confirm its protective effects in animal models (Li et al., 2021). This suggests quercetin’s potential to protect against oxidative damage by promoting autophagic pathways.

Quercetin’s ability to directly inhibit mTORC1 enhances its properties as an inducer of autophagy. Experimental evidence shows that quercetin promotes the nuclear translocation of TFEB, a master regulator of lysosomal biogenesis and autophagy, suggesting its role in enhancing cellular degradation mechanisms. The study also investigated the optimal dosage of quercetin for augmenting cellular degradation pathways. Cells treated with varying concentrations of quercetin ranging from 0.5 to 20 μM showed no significant differences in activity whether sourced from Sigma-Aldrich or USANA Health Sciences. Therefore, it is recommended that future studies consider using a dosage range of 0.5–20 μM for quercetin as an enhancer of cellular degradation pathways (Huang et al., 2018). Additionally, quercetin has been shown to activate AMPK, further influencing the mTOR pathway and autophagy induction (Xiao et al., 2022). This activation, along with mitochondrial membrane potential changes, suggests a complex interplay between quercetin, energy sensing, and autophagic regulation (Viollet et al., 2006; Dhanya et al., 2017). Research continues to uncover the breadth of quercetin’s impact on autophagy across various cell types and conditions. Its ability to modulate key signaling pathways like mTORC1 and AMPK underscores its therapeutic potential. However, the intricate mechanisms of quercetin’s action on autophagy demand further exploration to fully understand its benefits and applications in medical and clinical settings.

#### 3.2.3 Quercetin’s impact on necroptosis

Necroptosis, an inflammatory form of cell death, diverges from the orderly process of apoptosis, unfolding in response to extreme cellular distress caused by factors like trauma, infection, or toxic exposure (Zhang et al., 2018). This cell death pathway is marked by the uncontrolled rupture of the plasma membrane, leading to the release of cellular contents that can exacerbate inflammation (Golstein and Kroemer, 2007). Activation of necroptosis is mediated through specific receptors such as TLR-4, TLR3, TNFR, and Fas. These receptors, upon engagement, recruit adaptor proteins including FADD, TRADD, and TRIF, setting the stage for downstream signaling events culminating in necroptosis (Laster et al., 1988; Holler et al., 2000; Kaiser et al., 2013). Central to this pathway is RIPK1, which, under normal circumstances, is kept in check by ubiquitination. However, upon receiving death signals, RIPK1 is deubiquitinated, turning active and capable of initiating cell death (Feoktistova et al., 2011; Tenev et al., 2011). RIPK1, once activated, collaborates with RIPK3 to form the ripoptosome, a crucial assembly in determining cell fate towards necroptosis (Cho et al., 2009; Li et al., 2012; Wegner et al., 2017). The interaction between RIPK3 and MLKL leads to the formation of the necrosome, which migrates to the plasma membrane to form disruptive pores, furthering the necroptotic process (Cai et al., 2014; Wang et al., 2014; Dovey et al., 2018). Furthermore, cytosolic nucleic acid sensors like RIG-I and cGAS/STING, upon detecting foreign or abnormal nucleic acids, can trigger immune responses that inadvertently promote necroptosis, adding layers to the cell’s defensive mechanisms (Schock et al., 2017; Chen et al., 2018). Caspase-8, generally associated with apoptosis, plays a dual role by cleaving cytokine blockers like N4BP1 post-TNFR or TLR activation, thus facilitating cytokine release (Yang et al., 2018). Quercetin, known for its anti-inflammatory properties, has shown potential in modulating necroptosis by influencing the RIPK1 pathway. Studies indicate that quercetin can prevent the formation of necrosome complexes, thereby averting necroptosis and its associated inflammatory responses (Yu et al., 2022). By administering doses ranging from 50 to 200 μM, quercetin demonstrated an inhibitory effect on LPS-induced RIPK1 expression in chicken embryos, suggesting a protective role against necroptotic damage (Yu et al., 2022).

Moreover, quercetin can dampen the activation and nuclear translocation of NF-κB, a key regulator of inflammation, by targeting proteins in the NF-κB signaling pathway (Martínez-Flórez et al., 2005; Gao et al., 2020). Research by Susana Martínez-Flórez et al. demonstrated that quercetin could significantly reduce NO production in IL-1 stimulated rat liver cells by suppressing the expression of iNOS, likely through the inhibition of NF-κB activation, highlighting its therapeutic potential in inflammatory conditions. The study employed primary cultures of rat liver cells stimulated with IL-1 and treated with concentrations of quercetin ranging from 5 to 100 μmol/L. NO production was measured through the generation of reactive oxygen species and the release of nitrates into the culture medium. The researchers also analyzed the protein and mRNA levels of iNOS using Western blotting and reverse transcription-polymerase chain reaction, respectively. Furthermore, the activation of nuclear factor (NF)-κB and the levels of its inhibitors were assessed using electrophoretic mobility shift assay and Western blotting. The results suggest that quercetin suppresses NO production in IL-1 stimulated liver cells by inhibiting the expression of iNOS, likely through the suppression of IL-1 induced NF-κB activation (Martínez-Flórez et al., 2005). Recent research has discovered that quercetin supplementation in weaned pigs and the porcine intestinal epithelial cell line (IPEC-1) improved villus height in pigs following exposure to DON, enhanced intestinal barrier function, and inhibited necrotic signaling pathways. The study found that quercetin treatment, by suppressing the necrotic signal transduction pathway, offers potential protective effects against damage caused by exposure to deoxynivalenol (Liu et al., 2023). In essence, quercetin exhibits a protective mechanism against necroptosis by interfering with key signaling pathways and receptors involved in this cell death process. Its ability to inhibit RIPK1 activity, prevent necrosome formation, and reduce inflammatory responses positions quercetin as a promising natural metabolite for mitigating necroptotic cell death and inflammation in various pathological contexts.

#### 3.2.4 Quercetin’s modulation of pyroptosis

Pyroptosis, a lytic and pro-inflammatory form of cell death, is essential for combating infections and managing cellular stress, differentiating itself from other cell death mechanisms by its ability to invoke an inflammatory response (Jorgensen and Miao, 2015; Rathinam et al., 2019; Yu et al., 2021). It operates through two principal pathways: the canonical pathway, driven by caspase-1, and the non-canonical pathway, involving human caspase-4/5 or mouse caspase-11, both of which lead to the expulsion of inflammatory cytokines (Yu et al., 2021).

The initiation of pyroptosis via the canonical pathway starts with the detection of pathogenic or stress signals by pattern recognition receptors (PRRs), which sense pathogen-associated molecular patterns (PAMPs) or danger-associated molecular patterns (DAMPs). This detection triggers the assembly of inflammasomes, multiprotein complexes crucial for activating caspase-1 (Strowig et al., 2012; Liston and Masters, 2017). Various PRRs, such as NLRP1, NLRP3, NLRC4, AIM2, and pyrin, upon activation, collaborate with pro-caspase-1 and the adaptor protein ASC to form inflammasomes, which are instrumental in setting off pyroptosis (Fleshner, 2013; Sagulenko et al., 2013; Yang et al., 2019; Saadi et al., 2020). In this cascade, the activation of caspase-1 within the inflammasome complex leads to the cleavage of gasdermin-D (GSDMD) and the precursors of interleukin-1β (IL-1β) and interleukin-18 (IL-18), marking the cell for pyroptotic death (Martinon et al., 2002; Bergsbaken et al., 2009; Hornung et al., 2009; Sahoo et al., 2011; Zhao et al., 2018). The non-canonical pathway is characterized by the direct recognition of cytosolic lipopolysaccharide (LPS) by caspase-4/5 in humans or caspase-11 in mice, which also culminates in GSDMD cleavage and pyroptosis (Shi et al., 2014). Quercetin, a flavonoid with notable anti-inflammatory properties, has been identified as a potent modulator of pyroptosis. Studies have illustrated quercetin’s ability to inhibit the NLRP3 inflammasome pathway in THP-1 macrophages, reducing the maturation of N-terminal gasdermin-D (N-GSDMD) and IL-1β in a concentration-dependent manner (Luo et al., 2022). This inhibition is thought to occur through the suppression of P65 nuclear translocation and phosphorylation, alongside a reduction in reactive oxygen species (ROS) production (Luo et al., 2022). In experiments with LPS-primed mouse bone marrow-derived macrophages, quercetin administration resulted in a dose-dependent attenuation of NLRP3 and AIM2 inflammasome activation, highlighting its potential in reducing inflammatory responses (Domiciano et al., 2017). Additionally, quercetin’s influence extends to IFN-γ-primed keratinocytes, where it was found to suppress poly (dA:dT)-induced IL-18 secretion by downregulating the expressions of AIM2 and pro-caspase-1. This effect is attributed to the inhibition of JAK2 and STAT1 phosphorylation and their nuclear translocation, although high concentrations of quercetin were noted to negatively impact cell viability. The study recommends further research to determine the optimal dosage of quercetin, as the concentrations used in the study (5 and 10 mM) are relatively high (Lee et al., 2018). Quercetin’s effectiveness in controlling pyroptosis underscores its therapeutic potential in addressing inflammatory diseases and enhancing overall health. By targeting crucial components of the pyroptosis pathway, quercetin presents a promising natural solution for mitigating inflammation and preventing inflammatory cell death. However, further studies are essential to optimize quercetin’s dosage for maximal therapeutic benefits without adverse effects.

#### 3.2.5 Influence of quercetin on ferroptosis

Ferroptosis is a distinct, iron-dependent form of cell death, marked by excessive lipid peroxidation and oxidative stress, diverging from the genetically programmed nature of apoptosis. This cell demise mechanism is fueled by iron and entails the degradation of cellular lipids, leading to significant oxidative damage and metabolic disturbances (Dixon et al., 2012). Central to ferroptosis are the intricate processes of lipid, antioxidant, and iron metabolisms, each playing a pivotal role in the regulation and execution of this cell death pathway (Xiong et al., 2022; Zhou et al., 2022).

In the realm of lipid metabolism, the breakdown of lipid droplets through lipophagy releases polyunsaturated fatty acids (PUFAs), which are then activated by acyl-CoA synthetase long-chain family member 4 (ACSL4) to form PUFA-CoAs (Das, 2019). These fatty acids are further processed by lysophosphatidylcholine acetyltransferase 3 (LPCAT3), which incorporates PUFA-CoAs into phospholipids, contributing to the cellular lipid composition and influencing membrane dynamics and signaling (Liang et al., 2022). The oxidative stress facilitated by iron catalysis leads to the peroxidation of these PUFA-containing phospholipids, significantly compromising cell membrane integrity and ion homeostasis (Stockwell, 2022). Conversely, replacing PUFA-phospholipids with monounsaturated fatty acid (MUFA) counterparts serves as a protective mechanism against oxidative damage, stabilizing cell membranes (Magtanong et al., 2019).

Antioxidant metabolism plays a critical role in countering ferroptosis through the action of glutathione peroxidase 4 (GPX4), which utilizes glutathione (GSH) and selenium to detoxify lipid peroxides (Ursini and Maiorino, 2020; Miao et al., 2022). The System Xc-transporter, comprising subunits SLC7A11 and SLC3A2, is crucial for maintaining intracellular GSH levels by facilitating the exchange of extracellular cystine for intracellular glutamate (Li et al., 2022). In addition to the GPX4 pathway, alternative mechanisms such as FSP1-CoQ10 and GCH1-BH4 provide additional defense against lipid peroxidation, underscoring the multi-faceted nature of cellular protection against ferroptosis (Friedmann Angeli et al., 2019; Li et al., 2020).

Iron metabolism within cells involves the regulated uptake and storage of iron, with proteins such as transferrin, STEAP3, DMT1, and ferritin ensuring iron is appropriately managed to prevent oxidative damage. Excess ferrous iron (Fe2+) can catalyze the Fenton reaction, generating hydroxyl radicals that exacerbate lipid peroxidation, a hallmark of ferroptosis (Cheng et al., 2004; Sendamarai et al., 2008; Frazer and Anderson, 2014; Ji and Kosman, 2015).

Quercetin, a potent flavonoid, has emerged as a significant modulator of ferroptosis by influencing key aspects of this cell death pathway. It has been shown to mitigate the activation of lipid peroxidation and iron accumulation, thereby reducing the susceptibility of cells to ferroptosis-induced damage. Through its antioxidant properties, quercetin enhances the cellular defense against oxidative stress, potentially by stabilizing lipid membranes, regulating iron metabolism, and boosting the antioxidant capacity of cells (Shen et al., 2018; Henning et al., 2022). The intricate interplay between lipid oxidation, iron catalysis, and antioxidant defenses in ferroptosis underscores the potential of quercetin as a therapeutic agent. By targeting the molecular underpinnings of ferroptosis, quercetin offers a promising avenue for mitigating oxidative damage and preserving cellular integrity in conditions predisposed to iron-induced oxidative stress. Further research into the precise mechanisms by which quercetin influences ferroptosis will be crucial for harnessing its full therapeutic potential in combating diseases associated with oxidative damage and iron dysregulation.

Quercetin, a flavonoid with a notable affinity for iron, demonstrates significant efficacy in mitigating ferroptosis. Quercetin achieves this through its iron-chelating capabilities, particularly via its B ring catechol, which significantly reduces hydroxyl radical formation from metal-catalyzed reactions. In research by Evangelia Vlachodimitropoulou et al., it was highlighted that increasing quercetin concentration from 0 to 0.1 μM can increase the rate of cytosolic iron efflux by 2.5 times in control cells, and by 3.2 times following pre-loading with 10 μM Fe(II/III). Increasing the quercetin concentration to 1 or 5 μM significantly reduces the rate of cellular iron loss. Furthermore, the intake of flavonoids, including quercetin, may vary depending on dietary habits (Vlachodimitropoulou et al., 2011; Wang et al., 2021). Evangelia Vlachodimitropoulou’s research underscores quercetin’s dynamic role in modulating iron homeostasis. The study observed a 2.5- to 3.2-fold increase in cytosolic iron efflux in cells treated with increasing quercetin concentrations, highlighting its potential to alleviate iron overload (Vlachodimitropoulou et al., 2011).

Animal studies further corroborate quercetin’s protective effects against iron-induced oxidative damage and lipid peroxidation, showcasing its ability to safeguard organ function (Kim and Choe, 2018; Gholampour and Saki, 2019; Li et al., 2020). Dan Li et al.'s investigation into type 2 diabetes mellitus (T2DM) mouse models revealed quercetin’s capacity to restore glucose homeostasis and pancreatic β-cell integrity, attributing these effects to quercetin’s inhibition of pancreatic iron deposition and ferroptosis (Li et al., 2020).

Quercetin has also been credited with DNA protection in human colon carcinoma cells, illustrating its broader protective scope against iron overload (Glei et al., 2002). Moreover, it modulates the activity of the iron transporter ferroportin, possibly through microRNA interactions, suggesting a nuanced approach to iron regulation in the body. Marija Lesjak et al. provided insights into quercetin’s influence on intestinal iron absorption and ferroportin expression, demonstrating its dual role in modulating iron uptake and efflux. In the study, rats were treated with a dose of 50 mg/kg, and when treating Caco-2 cells, various concentrations of quercetin ranging from 0.1 to 10 mmol/L were used (Lesjak et al., 2014).

Quercetin’s antioxidant properties extend to the inhibition of lipoxygenase activity, reducing the uptake of oxygen and the peroxidation of linoleic acid, thereby curbing the generation of lipid peroxides. Wang et al.’s study further illustrates quercetin’s role in repressing ferroptosis in acute kidney injury scenarios by modulating ATF3 expression, system Xc-activity, GPX4 levels, and enhancing glutathione availability (Wang et al., 2021). Quercetin emerges as a multifaceted agent against ferroptosis, acting through iron chelation, antioxidant defense enhancement, and modulation of critical ferroptosis-related pathways. Its broad spectrum of activity not only highlights its potential in mitigating oxidative stress and lipid peroxidation but also underscores its therapeutic promise in conditions marked by iron overload and ferroptosis. Further exploration into quercetin’s mechanisms of action and optimal dosing will be vital for harnessing its full potential in preventing and treating ferroptosis-related diseases.

#### 3.2.6 Quercetin’s role in modulating cuproptosis

Cuproptosis, a novel type of cell death driven by copper accumulation, is emerging as a significant area of study within cellular biology. Unlike other forms of cell death, cuproptosis is distinguished by its reliance on copper, an essential yet potentially toxic trace element in cellular functions (Chen et al., 2022; Tsvetkov et al., 2022). The intricacies of cuproptosis involve the interaction of copper with cellular proteins, particularly through a process known as protein lipoylation. This post-translational modification, crucial for the activity of certain enzymes within the tricarboxylic acid (TCA) cycle, becomes a focal point for copper toxicity when excessive copper binds to the lipoyl groups, disrupting normal cellular processes (Solmonson and DeBerardinis, 2018).

Research by Tsvetkov et al. has underscored the vulnerability of cells with active TCA cycles and abundant lipoylated enzymes to copper-induced toxicity. The binding of copper to these enzymes precipitates protein aggregation, destabilizes iron-sulfur cluster proteins, and prompts a stress response, marking cells for cuproptosis (Tsvetkov et al., 2022; Zheng et al., 2022).

Quercetin, known for its antioxidant and metal-chelating properties, is under investigation for its potential to mitigate copper-induced cellular damage. Although detailed mechanisms by which quercetin affects cuproptosis remain to be fully elucidated, evidence suggests its capability to chelate copper ions, thereby reducing oxidative stress induced by copper overload (Lomozová et al., 2021). Studies exploring the interactions between quercetin metabolites and copper reveal that these metabolite can form complexes with copper, potentially attenuating copper-induced hemolysis, with isorhamnetin showing particular efficacy in this regard.

Moreover, quercetin has been shown to modulate copper’s effect on metallothionein, a protein involved in metal detoxification, in a dose-dependent manner. This modulation suggests quercetin’s role in enhancing the cellular response to copper toxicity, offering insights into its therapeutic potential for managing copper-related disorders. The research found that quercetin enhances the induction of metallothionein by copper in a dose-dependent manner and produces a cumulative effect after repeated exposures. Compared to a single treatment, the effective concentration of quercetin remains lower after repeated treatments. Just 10 µM of quercetin is sufficient to reduce metallothionein expression stimulated by zinc, and quercetin has no effect on cadmium-induced metallothionein (Kuo et al., 2001). The metabolite anti-inflammatory effects, possibly through the inhibition of NF-κB/IκBα and p38 MAPK signaling pathways, further contribute to its protective actions against copper-induced cellular stress (Wang et al., 2017). The therapeutic implications of quercetin in cuproptosis and copper toxicity are promising, yet necessitate further investigation to determine optimal dosages and to fully understand its mechanisms of action. The exploration of quercetin as a natural metabolite for counteracting copper-related cellular damage opens new avenues for the development of treatments for diseases characterized by copper dysregulation. With ongoing research, quercetin could potentially become a valuable tool in the modulation of cuproptosis and the management of copper-induced pathologies.

### 3.3 Quercetin’s influence on liver fibrosis and its role in traditional Chinese medicine (TCM)

Liver fibrosis, marked by the excessive buildup of fibrous tissue, arises as a consequence of chronic liver damage, often stemming from factors like viral hepatitis, alcohol abuse, non-alcoholic fatty liver disease (NAFLD), and autoimmune disorders (Poynard et al., 2003; Kondili et al., 2005; Yönem et al., 2006; Pais et al., 2013; Wu et al., 2014; Okura et al., 2015; Wu et al., 2019; Wang et al., 2020). This condition, characterized by a progressive alteration in liver architecture, can escalate to cirrhosis, liver failure, or even hepatocellular carcinoma if not adequately addressed (Roche et al., 2008; Sakurai and Kudo, 2013; Zhou et al., 2014). Understanding the pathogenesis of liver fibrosis is essential for developing effective treatments aimed at mitigating or reversing this fibrotic process.

The pivotal role in liver fibrosis development is attributed to hepatic stellate cells (HSCs), which reside in the liver sinusoids. These cells, upon activation by liver injury or inflammation, transform into myofibroblast-like cells that produce excessive amounts of extracellular matrix (ECM), leading to the fibrotic scarring of the liver (Brenner et al., 2000; Bekki et al., 2017; Zhang et al., 2017; Cai et al., 2018; Kiagiadaki et al., 2018). HSC activation is driven by both intracellular signals, such as the TGFβ/Smad, Notch, and Wnt/β-catenin pathways, and extracellular stimuli from other liver cells or external factors like cytokines and viral infections (Yan et al., 2021). This dual pathway of activation underscores the complex regulatory environment that promotes liver fibrosis.

Activated HSCs and fibroblasts are central to the fibrosis process, contributing to the pathological accumulation of collagen types I and III in the liver. This overproduction of collagen, a key component of the ECM, disrupts liver tissue architecture, impairs organ function, and obstructs blood flow (Musso et al., 1998; Du et al., 1999; Higashi et al., 2017; Ortiz et al., 2021). Quercetin, a flavonoid present in various TCM formulations, has demonstrated potential in addressing liver fibrosis. Through its multifaceted actions, quercetin targets the underlying mechanisms of fibrosis, offering a promising therapeutic avenue. Its potential to modulate HSC activity, reduce oxidative stress, and inhibit the excessive deposition of collagen marks quercetin as a significant metabolite in the fight against liver fibrosis.

**FIGURE 5:**
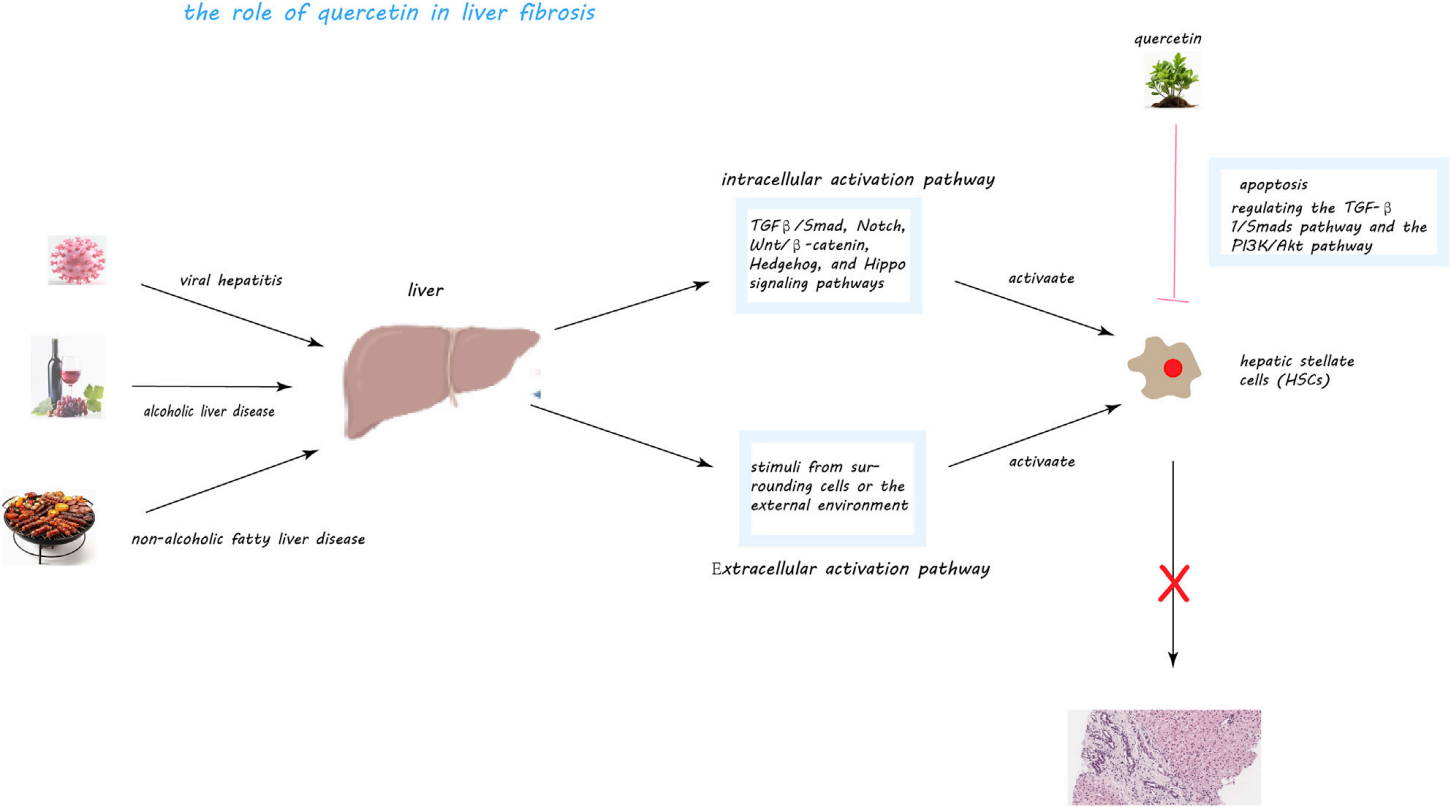
Quercetin’s Mechanisms in Mitigating Liver Fibrosis. This figure illustrates quercetin’s multifaceted role in combating liver fibrosis, a condition exacerbated by factors like viral hepatitis, alcoholic and non-alcoholic fatty liver disease (NAFLD), through the activation of hepatic stellate cells (HSCs). Central to fibrosis progression, HSCs, upon activation, produce an excess of extracellular matrix metabolites, including collagen, fostering fibrous scar tissue formation within the liver. Quercetin intervenes by inducing apoptosis in activated HSCs, thus diminishing their numbers and fibrogenic output. It notably inhibits the TGF-β1/Smads and PI3K/Akt pathways, crucial for HSC activation and collagen synthesis, and associated with cell survival and proliferation, respectively. Through these actions, quercetin effectively reduces HSC activation and their fibrogenic activity, offering a promising approach to slowing liver fibrosis progression.

While phenolic chemicals, as characteristic chemical structures of quercetin, are also widely present in other plants, and from the perspective of organic chemistry electronic effects, the benzene ring inherently involves the overlap of sp2 orbitals between carbon atoms when they come close to each other. Due to the close proximity of the carbon atoms, this further leads to the formation of side-by-side chemical bonds by the carbon atoms’ p orbitals vertical to the sp2 orbitals, resulting in a unique cyclic large pi-bond structure with 6 carbon atoms sharing 6 electrons. Therefore, according to the Huckel 4n+2 rule, this will possess aromaticity. This leads to benzene derivatives exhibiting unusual antioxidative properties, creating a paradox where other phenolic plants with benzene ring structures also possess similar chemical characteristics, suggesting that similar extracts could achieve similar effects. However, if we revisit the chemical formula of quercetin (Figure 1), we can see that while aromaticity brings hydrophobic interactions, a major driving force in chemical biology for protein folding and protein-ligand binding, the same hydrophobicity means that extracts with this structure cannot be absorbed through the small intestine and portal system. Considering most microbes reside in the large intestine, this implies that most phenolic plants are unlikely to be decomposed and absorbed by microbes. The presence of numerous hydroxyl functional groups in the chemical structure of quercetin means that although water molecules have a strong tendency to form hydrogen bonds, they cannot do so with the cyclic large pi-bond structure of the benzene ring, and one of the water molecule’s four charges will always face the benzene ring. Thus, to form as many hydrogen bonds as possible, water molecules will choose to rearrange, enveloping the entire benzene ring structure to form hydrophobic interactions, but the hydroxyl functional groups, which readily form hydrogen bonds, effectively break or alter the local arrangement of water molecules, thus forming hydrophilicity, making it easier to be absorbed through the small intestine. Moreover, in plants, quercetin’s hydroxyl functional groups are usually substituted by sugars, ethers, and phenolic acids (Materska, 2008), making quercetin with glycosides better at hydrophilizing, thereby increasing the likelihood of absorption by the body. Since liver cells require sugars for various biochemical reactions, when the sugar ligands are removed, the original hydrophobicity is restored. This clever structure gives quercetin characteristics different from other phenolic plants, and this property is likely specifically targeted towards the liver. Therefore, research into quercetin’s effects on liver fibrosis highlights its role in traditional Chinese medicine as a natural remedy with hepatoprotective properties. By interfering with the pathways that lead to HSC activation and collagen overproduction, quercetin contributes to the preservation of liver function and the prevention of fibrosis progression (Figure 5). Further exploration into quercetin’s therapeutic potential, within the context of TCM and beyond, could pave the way for novel interventions in liver fibrosis management, emphasizing the importance of early detection and treatment in curtailing the adverse outcomes associated with this condition.

Liver fibrosis emerges from an intricate web of signaling pathways and molecular interactions, underlining the complexity of its pathogenesis. A deep understanding of these mechanisms is pivotal for devising effective strategies for its prevention and treatment. Among the myriad pathways implicated, the Notch and JAK/STAT signaling pathways are of particular interest due to their significant roles in the progression of liver fibrosis. The Notch signaling pathway, integral to cell communication and various biological functions such as cell fate determination and differentiation, is implicated in liver fibrosis through its influence on hepatic stellate cells (HSCs) and collagen synthesis (Meliou et al., 2011; Espinoza et al., 2013). This pathway involves four receptors (Notch1-4) and five ligands (Dll1, Dll3, Dll4, Jagged1, and Jagged2), which orchestrate the cellular response during fibrosis (Shimizu et al., 2000; Briot and Iruela-Arispe, 2015). Notably, increased activity of Notch3 has been observed in fibrotic liver, promoting HSC activation and exacerbating fibrosis (Chen et al., 2012; Ni et al., 2018). The pathway also affects macrophage polarization, skewing the balance towards a pro-fibrotic environment (Bansal et al., 2015; Aimaiti et al., 2019).

Concurrently, the JAK/STAT signaling pathway, known for regulating cell growth and immune responses, has been identified as a key player in liver repair and fibrosis. It is activated by cytokines and growth factors like IL-6, IFN-γ, and TGF-β, directly influencing HSC transformation and fibrosis severity (Klein et al., 2005; Kong et al., 2012; Kagan et al., 2017; Tang et al., 2017; Xin et al., 2020). IL-6, for example, can induce HSCs to transform into myofibroblast-like cells, highlighting a potential target for therapeutic intervention.

Quercetin, a flavonoid present in various Traditional Chinese Medicine (TCM) formulations, has shown promise in targeting HSCs through these pathways, suggesting its potential in reversing liver fibrosis. Studies have demonstrated quercetin’s ability to inhibit HSC proliferation, induce apoptosis, and modulate significant pathways involved in fibrosis, such as TGF-β1/Smads and PI3K/Akt (He et al., 2016; Wu et al., 2017).

Research within the realm of TCM, particularly in China, has explored quercetin-containing decoctions for their anti-fibrotic effects. Formulations like Qiwei Qinggan Powder, Yinchenhao Decoction, and Fuzheng Huayu Recipe have been studied for their regulatory effects on key targets and signaling pathways, showing efficacy in reducing fibrosis and promoting liver health (Cai et al., 2019; Zhang et al., 2021; Hong et al., 2022; Huang et al., 2022; Sun et al., 2022; Zhang et al., 2022; Jiang et al., 2023).

While clinical trials specifically targeting liver fibrosis with TCM formulations containing quercetin are yet to be conducted, preliminary studies on quercetin’s impact on hepatocyte injury are promising. A randomized, placebo-controlled trial among Japanese subjects suggested improvements in liver function with quercetin-rich onion consumption (Nishimura et al., 2019). Furthermore, a phase I study indicated quercetin’s safety and potential antiviral activity in chronic hepatitis C patients (Lu et al., 2016) (Table 2 provides detailed listings of the dosages, duration of action, models, and other specific details of quercetin interventions in liver fibrosis mentioned in this paper).

## 4 Conclusion and perspectives

Quercetin, a polyphenolic flavonoid ubiquitously found in a variety of foods, offers a spectrum of health benefits, serving as the focus of this detailed exploration. Originating as a plant secondary metabolite, quercetin predominantly exists in glycosylated forms across numerous food sources, with onions, apples, berries, and leafy greens being particularly rich in this metabolite. Advances in extraction technologies, such as microwave-assisted and supercritical fluid extraction, have significantly surpassed traditional methods in efficiency and effectiveness, presenting a leap forward in quercetin utilization.

Despite its promising attributes, quercetin’s clinical application is hindered by its low oral bioavailability, attributed to challenges in absorption and rapid metabolic clearance. Innovations in dietary strategies and formulation technologies aim to enhance its bioavailability, ensuring a broader realization of quercetin’s health-promoting potential. The endeavor to amplify quercetin’s systemic availability is supported by extensive research, aiming to fully harness its therapeutic prospects.

Quercetin’s role extends to the modulation of various cellular death pathways, including but not limited to apoptosis, autophagy, necroptosis, pyroptosis, ferroptosis, and cuproptosis. It has shown efficacy in inducing apoptosis across a range of cancer cell lines, disrupting the balance between cell survival and death signals. The evidence presented herein underscores quercetin’s capacity to engage with multiple molecular mechanisms underlying cell death, supported by a robust body of scientific research.

The interaction between quercetin and liver fibrosis unveils another layer of its therapeutic potential. Exhibiting antioxidant, anti-inflammatory, and antitumor properties, quercetin emerges as a valuable agent in combating liver fibrosis. It achieves this through the regulation of critical signaling pathways, including NF-κB/IκBα, p38 MAPK, Bcl-2/Bax, TGF-β1/Smads, and PI3K/Akt. Moreover, the efficacy of Traditional Chinese Medicine (TCM) formulations containing quercetin against liver fibrosis has been highlighted, revealing promising outcomes in research studies.

This article serves as a detailed guide to quercetin, elucidating its sources, extraction methodologies, bioavailability challenges, and therapeutic roles in modulating cell death and combating liver fibrosis. Incorporating a wealth of research findings, this comprehensive overview not only illuminates the extensive benefits of quercetin but also emphasizes the ongoing efforts to enhance its bioavailability and therapeutic efficacy.

In summary, quercetin has been extensively validated in cellular and animal experiments, and future work should further explore the possibility of clinical trials to identify safe and effective dosages. Particularly, conducting more stringent, rigorous, and objective randomized double-blind controlled trials specifically targeting liver fibrosis will be a crucial step in the potential application of quercetin in the treatment of liver fibrosis. Overall, quercetin holds significant potential in the treatment of liver fibrosis and is worthy of further investigation.

## Figures and Tables

**Table:** 

**ACSL4**	Acyl-CoA Synthetase Long-Chain Family Member 4
**AMPK**	AMP-Activated Protein Kinase
**ASC**	Apoptosis-associated Speck-like Protein Containing a CARD
**CMA**	Chaperone-Mediated Autophagy
**DAMPs**	Danger-Associated Molecular Patterns
**DFCP1**	Double FYVE-Containing Protein 1
**DMT1**	Divalent Metal Transporter 1
**ECM**	Extracellular Matrix
**ER**	Endoplasmic Reticulum
**ETC**	Electron Transport Chain
**FADD**	Fas-Associated Death Domain
**FAS**	Fatty Acid Synthase
**FSP1**	Ferroptosis Suppressor Protein 1
**GCSH**	Glycine Cleavage System Protein H
**GPX4**	Glutathione Peroxidase 4
**GSDMD**	Gasdermin D
**GSH**	Glutathione
**HSCs**	Hepatic Stellate Cells
**IUPAC**	International Union of Pure and Applied Chemistry
**JAK**	Janus Kinase
**LAMP-2A**	Lysosome-Associated Membrane Protein 2A
**LC3**	Microtubule-associated Proteins 1A/1B Light Chain 3B
**LPCAT3**	Lysophosphatidylcholine Acyltransferase 3
**LPS**	Lipopolysaccharide
**MAE**	Microwave-Assisted Extraction
**MAPK**	Mitogen-Activated Protein Kinase
**MRP2**	Multidrug Resistance-Associated Protein 2
**MTORC1**	Mechanistic Target Of Rapamycin Complex 1
**NAFLD**	Nonalcoholic Fatty Liver Disease
**NASH**	Nonalcoholic Steatohepatitis
**NF-κB**	Nuclear Factor Kappa-light-chain-enhancer of Activated B Cells
**NLRP3**	NLR Family Pyrin Domain Containing 3
**PAMPs**	Pathogen-Associated Molecular Patterns
**PCD**	Programmed Cell Death
**PDH**	Pyruvate Dehydrogenase
**PI3K**	Phosphoinositide 3-Kinases
**PRRs**	Pattern Recognition Receptors
**PUFAs**	Polyunsaturated Fatty Acids
**RIPK**	Receptor-Interacting Protein Kinase
**ROS**	Reactive Oxygen Species
**SFE**	Supercritical Fluid Extraction
**SLC**	Solute Carrier Family
**STAT**	Signal Transducer and Activator of Transcription
**TCM**	Traditional Chinese Medicine
**TFEB**	Transcription Factor EB
**TGF-β**	Transforming Growth Factor Beta
**TLR**	Toll-Like Receptor
**TNF**	Tumor Necrosis Factor
**TCA**	Tricarboxylic Acid
**ULK1**	Unc-51 Like Autophagy Activating Kinase 1
